# Intrinsic Religious Motivation and Athlete Motivation from a Positive Psychology Perspective: Serial Mediation via Well-Being and Self-Confidence

**DOI:** 10.3390/bs16101911

**Published:** 2026-10-15

**Authors:** Özhan Can Yilmaz, İsa Kavalci, Nihal Akoğuz Yazici

**Affiliations:** Faculty of Sport Sciences, Recep Tayyip Erdoğan University, Rize 53100, Türkiye; ozhancan.yilmaz@erdogan.edu.tr (Ö.C.Y.); isa.kavalci@erdogan.edu.tr (İ.K.)

**Keywords:** licensed athlete, intrinsic religious motivation, psychological well-being, self-confidence, motivation

## Abstract

This study examined the serial mediating role of psychological well-being and self-confidence in the relationship between intrinsic religious motivation and athlete motivation. The study addresses a theoretical and empirical gap in the literature by explaining how religiosity-based spirituality is associated with motivational processes in athletes through a holistic serial mediation model encompassing multiple psychological mechanisms. A cross-sectional correlational survey design was employed. The sample consisted of 401 athletes (118 female, 283 male; *M*_age = 22.98 ± 4.8) actively competing in various sports disciplines across different regions of Turkey, each with a minimum of five years of licensed athletic experience. Data were collected using the Intrinsic Religious Motivation Scale, the Psychological Well-Being Scale, the Self-Confidence Scale, and the Intrinsic Motivation Scale. Serial mediation analysis was conducted using Hayes’s PROCESS macro (Model 6) with 5000 bootstrap resamples. The results indicated that intrinsic religious motivation was positively associated with psychological well-being (β = 0.221, *p* < 0.001) and self-confidence (β = 0.229, *p* < 0.001). Psychological well-being was positively associated with both self-confidence (β = 0.252, *p* < 0.001) and motivation (β = 0.307, *p* < 0.001), while self-confidence was positively associated with motivation (β = 0.141, *p* = 0.005). The total effect on motivation was significant (c = 0.171, *p* < 0.001); however, the direct effect became non-significant when mediators were included (c′ = 0.063, *p* = 0.194), revealing an indirect-only mediation pattern. All three specific indirect effects were significant: through psychological well-being (B = 0.060), through self-confidence (B = 0.029), and through the serial pathway (B = 0.007). These findings suggest that intrinsic religious motivation is associated with athlete motivation not directly but via the indirect pathways through psychological well-being and self-confidence.

## 1. Introduction

Religion and spirituality have been present across all societies throughout history, constituting fundamental structures that guide individuals’ lives. Although research on religion and spirituality indicates that religion is institution-centered whereas spirituality is meaning-centered, these concepts are frequently used interchangeably (Eryücel et al., 2024). Religion, as old as human history itself, is a fundamental component of human culture, encompassing elements such as establishing a relationship with a divine being superior to all humans, believing in a supernatural power, and worshipping God (Gafur et al., 2022; Wormley et al., 2023). According to data from the Pew Research Center (2025), approximately 76% of the world’s population identified with a religion as of 2020. Religious and spiritual practices have emerged as functional resources that support the integration of body, mind, and spirit in individuals’ daily lives (Stanford et al., 2023; J. Currier et al., 2024), thereby contributing to the sustainability of mental health at both individual and societal levels (Nolan, 2012; Garssen et al., 2020; Lucchetti et al., 2021).

In modern societies, individuals’ ability to maintain a meaningful life while preserving their psychological and mental integrity is closely associated not only with economic and social conditions (Galderisi et al., 2024) but also with internal value systems, belief structures, and existential orientations (Yaden et al., 2022; Zhang et al., 2023; Yücel et al., 2026). The secularization processes accompanying modernity have weakened the influence of traditional reference points in individuals’ identity construction and life-purpose determination (Bendle, 2002; Seleznev, 2020), leading individuals to turn to alternative spiritual resources in their search for meaning (Naim, 2013; Kosichenko, 2023). Within this framework, religious and spiritual orientation has come to be regarded as an important factor that fills this void by offering individuals meaning and a state of psychological well-being (Aisyahrani et al., 2024). Religious values are belief systems that shape individuals’ meaning-making processes, ethical orientations, and social relationships, providing guidance beyond material concerns through ideals such as compassion, love, morality, awareness, forgiveness, and commitment (Karimov et al., 2022; Ahmed, 2023; Sangma & Bharani, 2024). These values can serve as important psychological resources in individuals’ coping processes during stressful life events (Kolodinsky et al., 2008; Krause, 2019; Zou et al., 2020). It is well established that spiritual and religious values have influenced human cognition, emotions, behaviors, and consequently overall well-being as a metaphysical force since the earliest emergence of humanity (Tan & Yıldız, 2022). In particular, the relationship between religious and spiritual values and psychological factors has become an increasingly prevalent subject of research in recent years (Bożek et al., 2020; Hinterberger & Walter, 2025), with approximately 80% of studies in this domain focusing on the effects of religious-spiritual experience on individuals’ mental and psychological health (Garssen et al., 2020; Kao et al., 2020; Hannabuss, 2021).

Although the effects of spirituality on individuals’ psychological well-being have been addressed with increasing interest in the positive psychology literature and psychosocial health research (Ribeiro et al., 2021; Robina-Ramírez et al., 2021; Vieten & Lukoff, 2021; Baysal, 2022), the psychological mechanisms underlying these effects remain insufficiently elucidated (Milliman et al., 2017; Moon et al., 2022). A primary reason for this situation is that the discipline of psychology has been shaped within a secular and positivist paradigm for many years (Sami et al., 2021). This approach has led to the exclusion of religious and spiritual experiences from psychological research by categorizing them as subjective, irrational, and outside the scope of scientific understanding (Jensen, 2021). Another factor impeding deeper understanding in this area is the limited utilization of interdisciplinary approaches (Bouckaert & Zsolnai, 2012; Choe et al., 2023). Among the foremost of these underexplored domains are performance-oriented sport environments, which demand intense effort from both physical and mental perspectives (Roychowdhury, 2019; Noh & Shahdan, 2020; Guinto, 2020).

Athletes in professional sport environments characterized by high competition, intense expectations, and the ever-present risk of failure can sustain themselves not only through physiological competence but also through a robust psychological structure (Chrétien et al., 2024). In this context, the need for internal resources that enhance psychological resilience, develop mental capacity, and support motivational continuity has been increasingly emphasized in the sport psychology literature (Park & Jeon, 2023; Qin & Nazarudin, 2025). Therefore, identifying the religious and spiritual dimensions that can contribute to athletes’ psychological empowerment is important not only for their individual development but also for enhancing their performance in a sustainable manner (Upenieks et al., 2023; Shirvani et al., 2024; Sinden & Devall-Martin, 2024).

### 1.1. Conceptual Framework of the Study

This section examines the relational dynamics among intrinsic religious motivation, psychological well-being, self-confidence, and motivation, drawing upon frequently referenced theoretical approaches in literature. Psychological well-being is defined as an individual’s sense of meaning and purpose in life, self-actualization, capacity for positive relationships, and emotional satisfaction (Blasco-Belled & Alsinet, 2021; Basterra-Gortari et al., 2025). As positive psychology has increasingly emphasized in recent years, individuals’ psychological well-being is closely associated not only with external achievements or physical health (Vanden Poel & Hermans, 2019) but also with the meaning they attribute to their lives, their value orientations, and their states of internal commitment (García-Alandete, 2015; Waters & Fivush, 2015). Within this framework, it is proposed that spiritual and religious values can nourish performance-related psychological resources such as self-confidence and motivation by supporting psychological well-being. This relational mechanism has been explained through various theoretical perspectives, including meaning-centered theories, logotherapy, spiritual coping theory, and self-determination theory.

Frankl’s Meaning-Oriented Theory (Logotherapy) attributes the foundation of an individual’s psychological well-being to the capacity to find meaning in life (Frankl, 1985). According to this theory, psychological resilience in the face of challenging life events is directly linked to the meaning attributed to those experiences (Hornik, 2024). Frankl posited that the fundamental motive guiding human beings is neither the pursuit of pleasure nor power but the need to attribute meaning to one’s life. In this context, spiritual and religious values emerge as one of the principal internal resources supporting an individual’s search for meaning (Kolodinsky et al., 2008; Bharti & Verma, 2018). Logotherapy is particularly relevant to athletes, as it facilitates the reduction in psychological disintegration by enabling them to attribute meaning to stressful situations such as injury, failure, or intense performance pressure (Eryücel et al., 2024; Noh et al., 2024). In parallel, Pargament’s Spiritual Coping Theory proposes that individuals can enhance their psychological adjustment by turning to religious and spiritual resources during stressful life events (Pargament, 1997). In this regard, spirituality offers an effective coping strategy through functions such as strengthening the sense of control, restructuring threat perception, and maintaining emotional balance (Aggarwal et al., 2023; Desalegn et al., 2023). When evaluated from the perspective of athletes, spiritual coping strategies contribute to the regulation of anxiety and preservation of performance, particularly during competition processes characterized by uncertainty and pressure (Roychowdhury, 2019). Consequently, both theoretical perspectives indicate that spiritual and religious values constitute an important internal resource supporting athletes’ psychological well-being. Based on this evidence, the following hypothesis was formulated:

**H_1_.**  *Athletes’ levels of intrinsic religious motivation is positively associated with their psychological well-being.*

Self-Determination Theory (Deci & Ryan, 1985) posits that an individual’s psychological well-being depends on the satisfaction of three basic psychological needs: autonomy, competence, and relatedness. According to this theory, intrinsic motivation is shaped not solely by external rewards or social approval but by the capacity to lead a meaningful, authentic life consistent with one’s values. Numerous recent studies have demonstrated significant associations between the basic psychological needs of self-determination theory and spiritual experiences and values (Dierendonck, 2012; Chiang, 2024; Sviridova, 2024). Spirituality can contribute to the satisfaction of autonomy and competence needs by aligning individuals’ behaviors with a larger belief system, ethical principles, or life purpose (Wang et al., 2019; Khuan et al., 2023). From the perspective of athletes, spirituality supports the development of self-confidence grounded in self-actualization rather than merely achievement orientation, thereby strengthening psychological resilience and emotional stability (Watson & Nesti, 2005; Trigueros et al., 2019). Indeed, findings in the literature indicate that spiritual and religious values enhance self-confidence by nurturing athletes’ competence perceptions and sense of self-worth (Zardoshtian et al., 2018; Narimani et al., 2011). Therefore, Self-Determination Theory suggests that intrinsic religious motivation can serve as a powerful internal resource fostering self-confidence in athletes. In light of this evidence, the following hypothesis was developed:

**H_2_.**  *Athletes’ levels of intrinsic religious motivation is positively associated with their self-confidence.*

The positive psychology approach emphasizes that individuals’ well-being is not limited to subjective happiness but is also closely related to functional psychological resources such as self-confidence, intrinsic motivation, and psychological resilience (Chiesi et al., 2022; Zhao et al., 2022). Within this framework, individuals with higher levels of psychological well-being are reported to have more positive self-perceptions and stronger tendencies to direct their behaviors through internal drives (Puia et al., 2025). Empirical studies conducted in sport psychology similarly support this theoretical framework, demonstrating that athletes with higher psychological well-being exhibit more determined, motivated, and self-confident dispositions in performance processes (Peris-Delcampo et al., 2024; Singh et al., 2024). These findings suggest that psychological well-being should be regarded not merely as an outcome of performance but as a dynamic resource that nourishes performance-related psychological variables such as motivation and self-confidence. Furthermore, the relationship between self-confidence and motivation is strongly supported in the positive psychology and sport psychology literature. According to Bandura’s (1997) self-efficacy theory, individuals’ beliefs in their ability to successfully perform a given task directly influence their motivation toward that task. In the sport context, athletes with high self-confidence have been reported to set more ambitious goals, demonstrate greater resilience in the face of obstacles, and exhibit sustainable motivational patterns (Hays et al., 2009; Machida et al., 2012). This theoretical and empirical framework points to a sequential mechanism whereby psychological well-being nourishes self-confidence, which in turn feeds motivation.

It can be argued that levels of psychological well-being supported through spiritual and religious values constitute an important psychological resource that strengthens athletes’ self-confidence and, subsequently, their intrinsic motivation. The capacity of intrinsic religious motivation to support individuals’ search for meaning, self-regulation processes, and internal equilibrium can be considered a fundamental component of the holistic approach that positive psychology offers to sport psychology. Based on this theoretical framework, the following hypotheses were developed for testing in this study:

**H_3_.**  *Athletes’ psychological well-being is positively associated with their self-confidence.*

**H_4_.**  *Athletes’ psychological well-being is positively associated with their motivation.*

**H_5_.**  *Athletes’ self-confidence is positively associated with their motivation.*

When the theoretical framework and empirical findings presented above are evaluated collectively, it can be proposed that intrinsic religious motivation feeds athlete motivation not only directly but also through indirect pathways via psychological well-being and self-confidence. The logotherapy and spiritual coping literature demonstrates that intrinsic religious motivation strengthens psychological well-being by providing individuals with a sense of meaning and psychological resilience (Frankl, 1985; Pargament, 1997). Furthermore, the positive association of psychological well-being with both self-confidence and motivation has been supported by positive psychology and sport psychology research (Peris-Delcampo et al., 2024; Singh et al., 2024). Similarly, psychological resources such as self-regulation and goal orientation provided by intrinsic religious motivation have been reported to directly strengthen athletes’ self-confidence, which in turn positively influences motivation (Bandura, 1997; Zardoshtian et al., 2018; Hays et al., 2009).

This theoretical chain suggests that the association between intrinsic religious motivation and athlete motivation may operate through three complementary indirect pathways. First, intrinsic religious motivation may nourish motivation by strengthening psychological well-being. Second, intrinsic religious motivation may support motivation by enhancing self-confidence. Third, and theoretically the most comprehensive, a serial mediation pathway may operate whereby intrinsic religious motivation first elevates psychological well-being, which strengthens self-confidence, and this enhanced self-confidence in turn feeds motivation. This third pathway is derived from the integration of Self-Determination Theory’s basic psychological needs hierarchy (Deci & Ryan, 1985; Ryan & Deci, 2000) and Bandura’s (1997) self-efficacy theory. The sense of well-being nourished by intrinsic religious motivation enhances the individual’s competence perception and consequently their self-confidence, and the enhanced self-confidence sustains intrinsic motivation. It is important to note that although the broader concepts of spirituality, religiosity, and religion are discussed in the theoretical framework to situate the present research within the relevant literature, the construct directly measured in this study is intrinsic religious motivation, which refers to the internalization of religious beliefs as a primary motivational framework guiding behavior. Accordingly, the findings and their interpretation pertain specifically to intrinsic religious motivation rather than to spirituality or religiosity in their broader senses. Based on this framework, the following mediation hypotheses were developed:

**H_6_.**  *Psychological well-being mediates the relationship between intrinsic religious motivation and motivation.*

**H_7_.**  *Self-confidence mediates the relationship between intrinsic religious motivation and motivation.*

**H_8_.**  *Psychological well-being and self-confidence serially mediate the relationship between intrinsic religious motivation and motivation.*

### 1.2. Purpose and Significance of the Study

The purpose of this study was to examine the associations among licensed athletes’ intrinsic religious motivation levels, psychological well-being, self-confidence, and motivation, and to test the serial mediating role of psychological well-being and self-confidence in this relationship within the framework of a serial mediation model.

In recent years, a marked increase has been observed in studies examining the multidimensional effects of religiosity-based spirituality on individuals’ psychological functioning, with these effects being supported by empirical research findings (McMinn et al., 2009; Obregon et al., 2022; Bellehumeur et al., 2022; J. M. Currier et al., 2023; Garcia et al., 2023; Sandage, 2024).

Indeed, the literature demonstrates that spiritual and religious values play a functional role in the development of psychological constructs such as self-awareness (Burney et al., 2017), psychological well-being (Ellison & Fan, 2008; Vitorino et al., 2018), stress coping skills (Graham et al., 2001; Yun et al., 2019), and life satisfaction (Yaden et al., 2022), thereby positioning religious orientation as an important internal resource supporting individuals’ psychological resilience (Schwalm et al., 2021; Shabani et al., 2023; Howard et al., 2023). Accordingly, given the contributions of spirituality to individuals’ psychological resilience and well-being, examining its potential effects on the psychological processes of athletes who operate under high performance demands and stress emerges as a meaningful necessity from both theoretical and applied perspectives. The sustainability of performance in athletes is closely associated not only with physical competence (Gould et al., 2002; Prasetiyo et al., 2022) but also with psychological factors such as motivation, self-confidence, and psychological well-being (Liew et al., 2019; Pelin et al., 2020; Martín-Rodríguez et al., 2024). These constructs directly influence athletes’ attitudes during training and competition processes, representing key factors that determine both individual development and sport achievement (Park & Jeon, 2023; Jekauc et al., 2023; Patil, 2024; Krasmik et al., 2024).

The existing literature indicates that although studies examining the effects of religion and spirituality on general psychological functioning have increased, this relationship remains limited in the context of athletes operating in high-performance and high-stress environments. In particular, scientific knowledge regarding the pathway through which athletes’ intrinsic religious motivation interacts with performance-related psychological variables such as psychological well-being, self-confidence, and motivation is insufficient. The majority of existing studies have examined bivariate correlations among these variables; however, the question of through which sequential psychological processes intrinsic religious motivation is associated with athlete motivation has remained unanswered. The present study aims to address this gap by testing whether intrinsic religious motivation is associated with motivation through the serial mediation of psychological well-being and self-confidence, thereby providing an evidence-based foundation for the development of future psychological support programs for athletes.

## 2. Materials and Methods

### 2.1. Research Design

**Figure 1:**
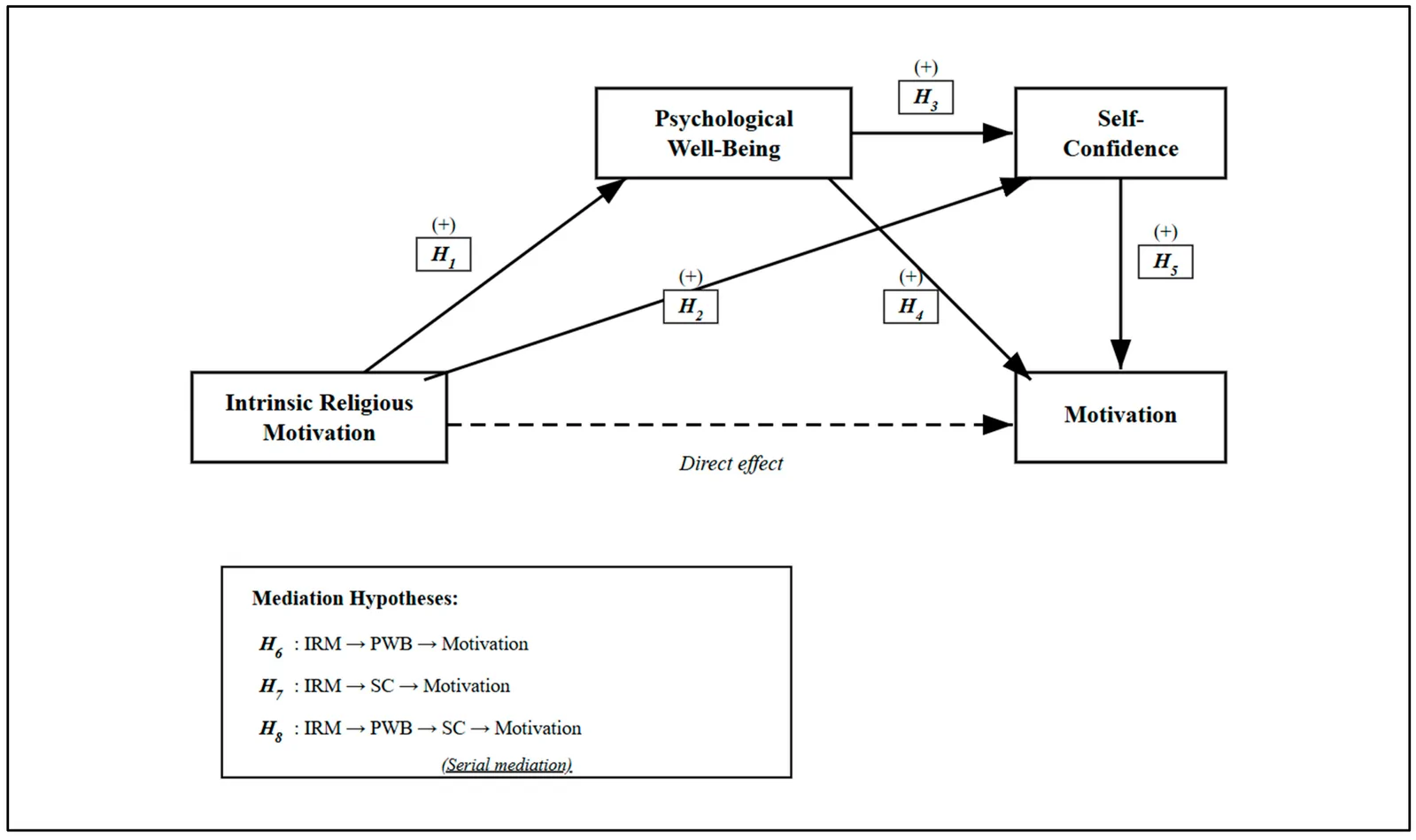
Research Model. *Note:* Solid arrows represent hypothesized direct paths; the dashed arrow represents the direct effect ($c^{\prime }$). (+) indicates expected positive associations.

This study employed a correlational survey design, a quantitative research approach, to identify the relationships among variables (Creswell & Creswell, 2018). The theoretical framework of the study was directed toward examining the direct effects of athletes’ intrinsic religious motivation levels on psychological well-being, self-confidence, and motivation, and the serial mediating role of psychological well-being and self-confidence in this relationship. The research model developed within this scope is presented in Figure 1.

### 2.2. Participants, Procedure, and Ethics

In this study, data on participants’ age and sex were collected. To examine the effects of athletes’ intrinsic religious motivation levels on psychological well-being, self-confidence, and motivation, the Intrinsic Religious Motivation Scale, the Psychological Well-Being Scale, the Self-Confidence Scale, and the Intrinsic Motivation Scale were administered. Data were collected both online via Google Forms and through face-to-face paper-and-pencil administration. Of the 401 participants, 278 (69.3%) completed the questionnaire online via Google Forms, while 123 (30.7%) responded through face-to-face paper-and-pencil administration. To examine potential measurement variance across administration modes, independent-samples *t*-tests were conducted on the composite scores of all study variables; no statistically significant differences were observed between the two groups, suggesting that the mode of administration did not introduce systematic bias. The sample comprised 170 (42.4%) professional and 231 (57.6%) amateur athletes across various sport disciplines. At the outset of the data collection process, participants were provided with detailed information regarding the purpose, scope, and content of the study, and it was explicitly stated that participation was entirely voluntary. Informed consent was obtained from all participants, and the process was conducted in accordance with ethical principles. The inclusion criteria were defined as being 18 years of age or older, having a minimum of five years of licensed athletic experience, and agreeing to participate voluntarily. The exclusion criteria were defined as not holding an active sports license, being under 18 years of age, and not consenting to voluntary participation. Convenience sampling was employed for sample selection in this study. The sample consisted of 401 actively licensed athletes (118 female, 283 male; *M*_age = 22.98, *SD* = 4.80) competing in various sports branches across different regions of Türkiye.

Prior to data collection, an a priori power analysis was conducted using G*Power 3.1.9.7 software to determine the minimum required sample size. The analysis was performed for linear multiple regression with three predictors (corresponding to the most complex equation in the serial mediation model), a medium effect size (*f*^2^ = 0.15), a significance level of α = 0.05, and a statistical power of 95% (1 − β = 0.95). The analysis yielded a minimum required sample size of 119 participants. The final sample of 401 athletes substantially exceeds this threshold, confirming adequate statistical power for the serial mediation analysis. Ethical approval for the study was granted by the Recep Tayyip Erdoğan University Social and Human Sciences Ethics Committee (Decision Date: 18 June 2025, Decision No: 2025/451).

### 2.3. Data Collection Instruments

A demographic information form including items on age and sex was used to identify the demographic characteristics of the participants. In addition, in line with the objectives of the study, the Intrinsic Religious Motivation Scale, the Psychological Well-Being Scale, the Self-Confidence Scale, and the Intrinsic Motivation Scale all of which have previously demonstrated validity and reliability were employed to assess participants’ levels of intrinsic religious motivation, psychological well-being, self-confidence, and intrinsic motivation, respectively.

#### 2.3.1. Intrinsic Religious Motivation Scale

The Intrinsic Religious Motivation Scale (IRMS) was developed by Hoge (1972) on the basis of the intrinsic religious orientation dimension of the Religious Orientation Scale originally devised by Allport and Ross (1967). The Turkish adaptation and psychometric evaluation were conducted by Karaca (2001). The IRMS has a unidimensional structure and consists of 10 items, of which 7 are positively worded and 3 are negatively worded; the latter are reverse-scored. Items are rated on a 5-point Likert-type scale (1 = strongly disagree, 5 = strongly agree), with total scores ranging from 10 to 50. Higher scores indicate greater levels of intrinsic religious motivation. Regarding reliability, the Cronbach’s alpha coefficient was reported as 0.84 in the original adaptation study and was calculated as α = 0.82 in the present study. Construct validity analyses indicated that the unidimensional structure of the scale was supported by confirmatory factor analysis (CFA), with acceptable model fit indices. These findings demonstrate that the IRMS is a valid and reliable instrument for measuring intrinsic religious motivation.

#### 2.3.2. Psychological Well-Being Scale

The Psychological Well-Being Scale was used to assess participants’ levels of psychological well-being. The scale was developed by Diener et al. (2010) to evaluate socio-psychological well-being, and its Turkish adaptation, validity, and reliability studies were conducted by Telef (2013). Exploratory factor analysis revealed a unidimensional structure that accounted for 42% of the total variance, with item factor loadings ranging from 0.54 to 0.76. Confirmatory factor analysis results indicated that the model exhibited acceptable fit indices. Regarding reliability, the Cronbach’s alpha coefficient was calculated as 0.80, and test–retest reliability demonstrated a high, positive, and significant correlation between the two administrations (*r* = 0.86, *p* < 0.001). In the present study, the Cronbach’s alpha coefficient was α = 0.77. The scale comprises 8 items rated on a 7-point Likert-type scale (1 = *strongly disagree*, 7 = *strongly agree*). Higher total scores indicate higher levels of psychological well-being. These findings indicate that the scale is a valid and reliable instrument for measuring individuals’ psychological well-being. It should be noted that this instrument corresponds to Diener et al.’s (2010) Flourishing Scale, which assesses socio-psychological flourishing (perceived success in relationships, self-esteem, purpose, and optimism), and is conceptually distinct from Ryff’s (1989) six-dimensional eudaimonic model of psychological well-being.

#### 2.3.3. Self-Confidence Scale

The Self-Confidence Scale was used to assess participants’ self-confidence levels. The scale was developed by Alacapınar (2022) and aims to measure individuals’ general self-confidence levels. It consists of 11 items rated on a 7-point Likert-type scale (1 = *strongly disagree*, 7 = *strongly agree*), with total scores ranging from 11 to 77. Higher scores indicate greater levels of self-confidence. Regarding construct validity, exploratory factor analysis yielded a Kaiser–Meyer–Olkin (KMO) value of 0.794, and Bartlett’s test of sphericity was statistically significant. Factor analysis findings indicated that the scale accounted for 53.23% of the total variance, and item factor loadings were reported to be at acceptable levels. In terms of reliability, the Cronbach’s alpha internal consistency coefficient was calculated as 0.816, indicating a high level of reliability. In the present study, the Cronbach’s alpha coefficient was α = 0.70. These findings demonstrate that the Self-Confidence Scale is a valid and reliable instrument for measuring individuals’ self-confidence levels. The Alacapınar (2022) scale measures general self-confidence rather than sport-specific confidence (cf. Vealey’s Sport Confidence Inventory). This choice was consistent with the study’s focus on broad psychological mechanisms linking religiosity to motivation.

#### 2.3.4. Intrinsic Motivation Scale

The final 4-item form of the Intrinsic Motivation Scale was used to assess participants’ intrinsic motivation levels. The scale is based on the intrinsic motivation measure developed by Kuvaas and Dysvik (2009), and its Turkish validity and reliability study was conducted by Aydemir Dev et al. (2022). The final form has a unidimensional structure comprising 4 items rated on a 5-point Likert-type scale (1 = *strongly disagree*, 5 = *strongly agree*). Confirmatory factor analysis results for construct validity indicated that the 4-item structure demonstrated a high level of model fit, and the obtained fit indices revealed good model fit values. Regarding reliability, the Cronbach’s alpha internal consistency coefficient was reported above 0.80, while in the present study it was calculated as α = 0.67. These findings support the scale as a valid and reliable instrument for measuring intrinsic motivation. The Kuvaas and Dysvik (2009) measure was originally developed for work contexts; however, its items assess domain-general intrinsic motivation (e.g., enjoyment and satisfaction derived from one’s primary activity) rather than work-specific content, supporting their applicability to athletes whose sport constitutes their primary performance domain.

### 2.4. Data Analysis

The raw data obtained in this study were first checked and organized in Microsoft Excel, and subsequently transferred to IBM SPSS Statistics 27, JASP 0.19.3.0, and AMOS 24 for statistical analyses. Confirmatory factor analysis was performed separately for each scale as a single-factor model using AMOS 24 software to verify the unidimensional structure and construct validity of the individual measurement instruments prior to computing composite scores for the path analysis. Additionally, Minitab 20 Statistical Software was used for the visual analysis of descriptive findings and distributional properties. Data obtained from *N* = 408 participants were initially examined, and 7 cases were identified as univariate outliers based on standardized z-scores exceeding |z| > 3.29 (*p* < 0.001, two-tailed) on at least one composite variable, supplemented by visual inspection of boxplots (Tabachnick & Fidell, 2013). These cases were excluded, and statistical analyses were performed on data from *n* = 401 participants, constituting 98.3% of the initial sample.

In the preliminary analyses, the suitability of the dataset for parametric analyses and path analysis was assessed through descriptive statistics and assumption tests. Normality of the variables was examined through skewness and kurtosis coefficients, with the ±2 range proposed by George and Mallery (2010) adopted as the acceptable threshold. To evaluate the assumptions of linearity and multicollinearity, Pearson correlation coefficients among variables were examined; coefficients below 0.90 provided a preliminary indication of the absence of multicollinearity (Tabachnick & Fidell, 2013). Additionally, variance inflation factor (VIF) and tolerance values were computed for each regression equation in the serial mediation model. In **the regression equation** predicting self-confidence, VIF values for intrinsic religious motivation and psychological well-being were both 1.05 (tolerance = 0.95). In **the regression equation** predicting athlete motivation, VIF values ranged from 1.11 to 1.16, with tolerance values ranging from 0.86 to 0.90. All VIF values were well below the recommended threshold of 5, and all tolerance values exceeded 0.20, confirming the absence of multicollinearity among the predictor variables (Hair et al., 2019). Bivariate linear relationships among the main study variables were examined using Pearson product-moment correlation coefficients. The internal consistency reliability of the measurement instruments was assessed using both Cronbach’s alpha (α) and McDonald’s omega (ω) coefficients; values in the 0.60–0.70 range and above were considered acceptable for short scales (Tavakol & Dennick, 2011). To evaluate the convergent validity of the constructs, Composite Reliability (CR) and Average Variance Extracted (AVE) values were calculated. In cases where AVE values fell below the 0.50 threshold, a CR value exceeding 0.70 was considered sufficient for convergent validity (Fornell & Larcker, 1981; Hair et al., 2019).

The adequacy of the measurement models was evaluated based on multiple fit indices. In this regard, the chi-square to degrees of freedom ratio (χ^2^/df), the Root Mean Square Error of Approximation (RMSEA), the Comparative Fit Index (CFI), and the Standardized Root Mean Square Residual (SRMR) were considered. For acceptable model fit, the criteria of χ^2^/df < 5, RMSEA < 0.08, and CFI > 0.90 were adopted (Mishra & Datta, 2011; Schreiber et al., 2006). To test the hypothesized model, the serial mediating roles of psychological well-being and self-confidence in the relationship between intrinsic religious motivation and motivation were analyzed using the PROCESS Macro (Model 6) developed by Hayes (2022). For the evaluation of direct and indirect effects, a bootstrap resampling procedure with 5000 iterations was applied, and bias-corrected 95% confidence intervals were used. A confidence interval that did not contain zero was interpreted as indicating that the indirect effects were statistically significant (Hayes, 2022). Because all variables were collected via self-report questionnaires administered at a single time point, Harman’s single-factor test was conducted to assess the potential for common method variance (Podsakoff et al., 2003). All 33 items from the four scales (IRMS, FS, SCS, and SMS-II) were entered into an unrotated principal component analysis.

## 3. Results

### 3.1. Preliminary Analyses

Descriptive statistics, reliability coefficients, and inter-variable correlations for the variables used in the study are presented in Table 1.

Descriptive statistics, reliability coefficients, and intercorrelations for the study variables are presented in Table 1. Skewness (−0.59 to −0.12) and kurtosis (−0.97 to 0.01) values for all variables fell within the recommended thresholds, confirming that the data met the normality assumption. Regarding internal consistency reliability, the intrinsic religious motivation (α = 0.82, ω = 0.85) and psychological well-being (α = 0.77, ω = 0.78) scales demonstrated values above the acceptable level, whereas the self-confidence (α = 0.70, ω = 0.69) and motivation (α = 0.67, ω = 0.64) scales yielded borderline yet acceptable values for short scales (Tavakol & Dennick, 2011). Composite reliability (CR) values exceeded 0.79 for all scales. Average variance extracted (AVE) values for the Psychological Well-Being (0.40), Self-Confidence (0.30), and Intrinsic Religious Motivation (0.42) scales fell below the 0.50 threshold. However, the literature indicates that convergent validity can be considered acceptable when CR values exceed 0.70 (Fornell & Larcker, 1981; Hair et al., 2019). The CR values for all constructs (0.79–0.87) substantially met this criterion. Furthermore, when evaluating scale reliability, the small number of items was taken into consideration, and Cronbach’s α (0.67–0.82) and McDonald’s ω (0.64–0.85) values were found to be within acceptable limits for the research model (Tavakol & Dennick, 2011). Additionally, the Heterotrait-Monotrait (HTMT) ratio was computed for all construct pairs; all values fell below the conservative 0.85 threshold (Henseler et al., 2015): IRM–PWB = 0.304, IRM–SC = 0.452, IRM–MOT = 0.282, PWB–SC = 0.542, PWB–MOT = 0.513, SC–MOT = 0.566. The Fornell-Larcker criterion was also satisfied for all pairs, collectively supporting adequate discriminant validity.

Pearson correlation analysis revealed positive and statistically significant relationships among all variables (*r* = 0.17–0.36, *p* < 0.01). The strongest correlation was observed between psychological well-being and motivation (*r* = 0.36), whereas the weakest correlation was between intrinsic religious motivation and motivation (*r* = 0.17), a pattern that supports the possibility that this relationship operates indirectly through the mediating variables. All correlation coefficients remained below 0.90, indicating the absence of multicollinearity (Tabachnick & Fidell, 2013). The Harman’s single-factor test indicated that the first unrotated component accounted for 19.90% of the total variance, well below the 50% threshold (Podsakoff et al., 2003), suggesting that common method bias does not pose a serious threat to the validity of the findings.

As shown in Table 2, the CFA results indicated that all scales demonstrated acceptable model fit indices. However, it should be noted that the GFI value for the Self-Confidence Scale (0.87) fell slightly below the conventional threshold of 0.90, while the remaining fit indices (CFI = 0.90, RMSEA = 0.05, IFI = 0.90, PNFI = 0.78) met the acceptable criteria. Given that the GFI is known to be sensitive to sample size and model complexity, and its use as a primary fit criterion has been increasingly questioned in the methodological literature (Sharma et al., 2005; Hu & Bentler, 1999), the overall pattern of fit indices was considered sufficient to support the factorial validity of the scale. Additionally, the PNFI value for the Intrinsic Motivation Scale (0.33) reflects the mathematical constraint imposed by the scale’s brevity (4 items, df = 2), which limits the maximum achievable PNFI value regardless of model quality, rather than indicating inadequate model fit.

### 3.2. Direct Paths

**Figure 2:**
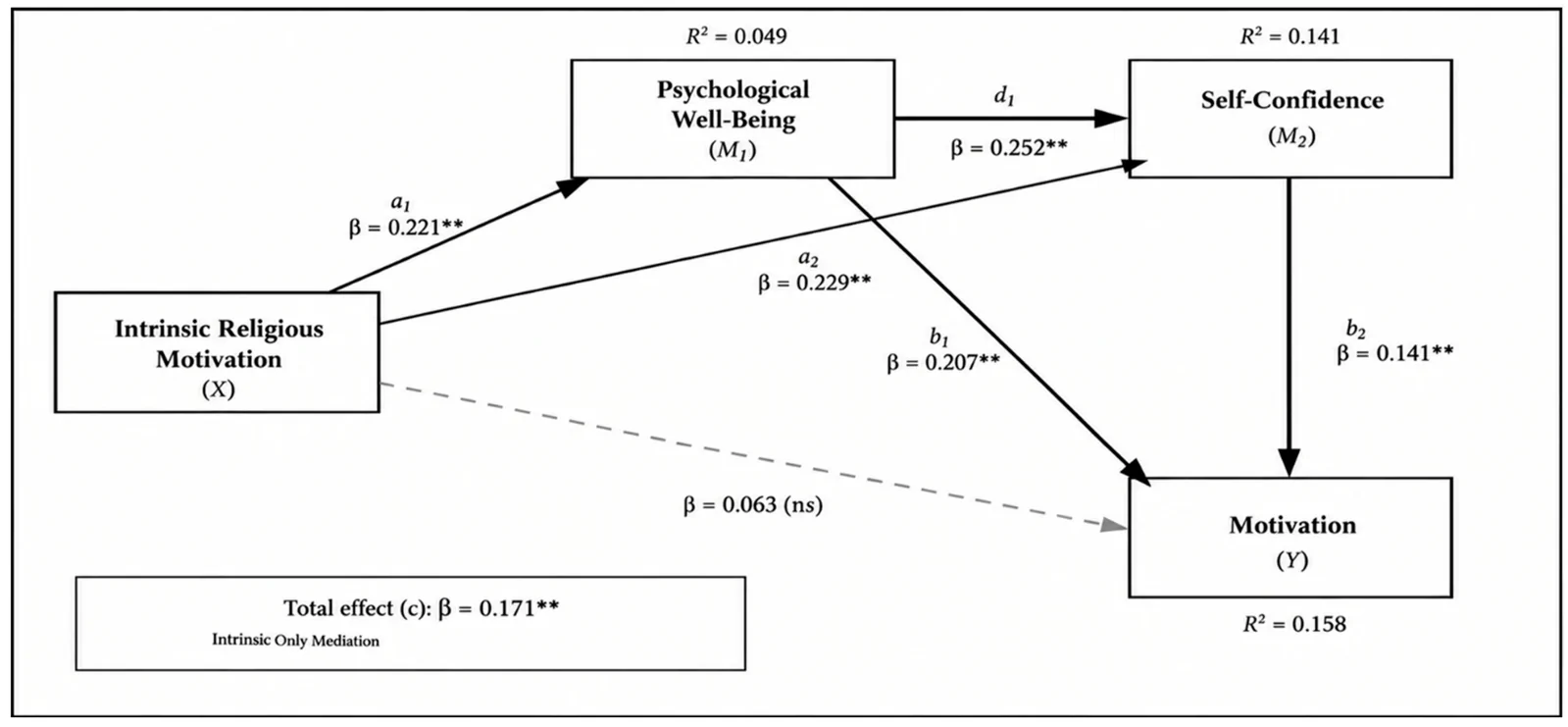
Serial Mediation Model of the Associations Among Intrinsic Religious Motivation, Psychological Well-Being, Self-Confidence, and Motivation. ***Note***. Standardized path coefficients (β) are shown. Solid lines represent statistically significant paths; the dashed line represents the non-significant direct effect (c′). Serial mediation was tested using Hayes (2022) PROCESS Model 6 with 5000 bootstrap resamples and bias-corrected 95% confidence intervals. *N* = 401. ** *p* < 0.01. ns = non-significant.

A serial mediation analysis was conducted using the PROCESS macro (Model 6; Hayes, 2022) to examine the serial mediating roles of psychological well-being and self-confidence in the relationship between intrinsic religious motivation and motivation. The model was estimated with 5000 bootstrap resamples, and the significance of indirect effects was evaluated using bias-corrected 95% confidence intervals (BC 95% CI). **A summary of hypothesis testing results for direct and indirect paths is presented in** Table 3, and standardized path coefficients are presented in Figure 2. The total effect of intrinsic religious motivation on motivation was statistically significant (c = 0.171, *p* < 0.001). However, when the two mediators were included in the model, the direct effect of intrinsic religious motivation on motivation became nonsignificant (c′ = 0.063, *p* = 0.194). The nonsignificance of the direct effect (c′) is consistent with an indirect-only mediation pattern (Zhao et al., 2010; Hayes, 2022), suggesting that, within the specified model, the statistical association between X and Y is conveyed via the indirect pathways through M_1_ and M_2_ rather than through a direct path.

Examination of the individual paths revealed that intrinsic religious motivation was positively and significantly associated with psychological well-being (*a*_1_ = 0.221, *p* < 0.001) and self-confidence (*a*_2_ = 0.229, *p* < 0.001). Psychological well-being, in turn, was positively and significantly associated with both self-confidence (*d*_21_ = 0.252, *p* < 0.001) and motivation (*b*_1_ = 0.307, *p* < 0.001). Self-confidence was also positively and significantly associated with motivation (*b*_2_ = 0.141, *p* = 0.005). Among the direct effects in the model, only the direct effect of intrinsic religious motivation on motivation (c′) was statistically nonsignificant.

### 3.3. Indirect Paths

Three specific indirect effects were examined in the model, and all were found to be statistically significant as their bootstrap confidence intervals did not contain zero. Specifically, the first indirect path through psychological well-being (H6: B = 0.060, Boot *SE* = 0.018, BC 95% CI [0.028, 0.098]), the second indirect path through self-confidence only (H7: B = 0.029, Boot *SE* = 0.013, BC 95% CI [0.006, 0.057]), and the third indirect path through both mediators in sequence (H8: B = 0.007, Boot *SE* = 0.004, BC 95% CI [0.001, 0.016]) were all statistically significant. The total indirect effect (B = 0.096, Boot *SE* = 0.023, BC 95% CI [0.054, 0.143]) was significant and accounted for the majority of the total effect (β = 0.171). These findings are consistent with an indirect-only mediation pattern (Zhao et al., 2010), suggesting that the statistical association between intrinsic religious motivation and motivation within the specified model is conveyed primarily through the indirect pathways involving psychological well-being and self-confidence.

The model explained 4.9% of the variance in psychological well-being (*R*^2^ = 0.049), 14.1% of the variance in self-confidence (*R*^2^ = 0.141), and 15.8% of the variance in motivation (*R*^2^ = 0.158). Notably, the inclusion of psychological well-being and self-confidence as serial mediators increased the explained variance in motivation from 2.9% (in the model with intrinsic religious motivation as the sole predictor) to 15.8%. The incremental contribution of psychological well-being corresponded to a small-to-medium effect size (Cohen’s *f*^2^ = 0.100), while the incremental contribution of self-confidence corresponded to a small but significant effect size (Cohen’s *f*^2^ = 0.020).

## 4. Discussion

This study examined the associations among intrinsic religious motivation, psychological well-being, self-confidence, and athletes’ motivation through a serial mediation framework, within the broader context of positive psychology and sport psychology. The findings indicated that the statistical association between intrinsic religious motivation and motivation was consistent with the hypothesized serial mediation model, with all indirect pathways through the proposed mediating variables reaching statistical significance.

The findings indicate that intrinsic religious motivation was positively and significantly associated with psychological well-being (H_1_: β = 0.221, *p* < 0.001). This finding is consistent with the positive psychology literature, which posits that spirituality contributes to the process of imbuing life with meaning, thereby enhancing stress-coping capacity and strengthening emotional balance. The contribution of spirituality to meaning-making processes has been one of the foundational tenets of psychology literature since Viktor Frankl’s logotherapy theory (Frankl, 1963). Indeed, according to Krok (2015), the search for meaning and religion–spirituality play significant roles in human life, and these two processes interact with each other. Religion and spirituality enhance individuals’ stress-coping capacity by adding meaning to their lives, which in turn contributes to their psychological adjustment. The effect of religion and spirituality on stress coping is further supported by the comprehensive meta-analysis by Ano and Vasconcelles (2005) and demonstrates that possessing spirituality significantly increases psychological well-being. Their findings indicated that spirituality enhances psychological well-being by reducing emotional disturbances and mitigating the effects of stress, while also demonstrating that spiritual well-being is directly and positively associated with individuals’ capacity to adapt to emotional burdens. Although these findings clearly demonstrate the psychological benefits of spirituality, it should not be overlooked that spirituality does not always produce positive outcomes. As noted by Mancini et al. (2023), negative spiritual coping strategies such as blaming God or struggling with one’s faith can exacerbate psychological distress. This suggests that the psychological impact of spirituality is closely related to its content and how it is experienced by the individual.

The findings also supported the hypothesized relationships between the mediating variables and the outcome variable. Psychological well-being was positively associated with both self-confidence (H_3_: β = 0.252, *p* < 0.001) and motivation (H_4_: β = 0.307, *p* < 0.001), and self-confidence was also positively associated with motivation (H_5_: β = 0.141, *p* = 0.005). Additionally, intrinsic religious motivation was directly and positively associated with self-confidence (H_2_: β = 0.229, *p* < 0.001). These findings are consistent with the existing literature. Zardoshtian et al. (2018) found that spirituality had a significant and direct effect on karate athletes’ sport self-confidence and exercise self-efficacy. Similarly, Narimani et al. (2011) reported that the intensity of religious and spiritual feelings predicted self-confidence by reducing competitive anxiety, suggesting that spirituality provides a cognitive tool for anxiety management. Regarding the relationship between self-confidence and motivation, the present finding is congruent with Bandura’s (1997) self-efficacy theory, which posits that individuals’ beliefs in their ability to succeed directly influence their motivation. Furthermore, Coussens et al. (2025) noted that self-confidence has a strong relationship with psychological well-being.

As a notable finding, the total effect of intrinsic religious motivation on motivation was significant (c = 0.171, *p* < 0.001); however, the direct effect became nonsignificant when the mediating variables were included in the model (c′ = 0.063, *p* = 0.194), indicating indirect-only mediation (Zhao et al., 2010). All three specific indirect effects were statistically significant: the path through psychological well-being alone (B = 0.060, 95% CI [0.028, 0.098]), the path through self-confidence alone (B = 0.029, 95% CI [0.006, 0.057]), and the serial path through both mediators in sequence (B = 0.007, 95% CI [0.001, 0.016]). Based on the standardized coefficients, the proportion of the total effect mediated was approximately 63% [(β total − β direct)/β total = (0.171 − 0.063)/0.171 ≈ 0.63]. These results suggest that, within the specified model, the statistical association between intrinsic religious motivation and athletes’ motivation is conveyed indirectly through psychological well-being and self-confidence. Roychowdhury’s (2019) systematic review supports this interpretation, reporting that spiritual–religious feelings are associated with athletes’ motivational processes primarily through intrinsic motivation, linking sport to personal growth and higher purposes beyond extrinsic rewards. The present findings extend this understanding by specifying the indirect pathways through which this statistical association is observed within the tested model.

## 5. Conclusions

This study suggests that intrinsic religious motivation is meaningfully associated with athletes’ psychological lives and is indirectly associated with motivation via the serial mediation of psychological well-being and self-confidence. All five direct path hypotheses (H_1_–H_5_) and all three indirect effect hypotheses (H_6_–H_8_) were supported. When the mediating variables were included in the model, the direct effect of intrinsic religious motivation on motivation became nonsignificant, and an indirect-only mediation pattern was observed (Zhao et al., 2010).

The serial mediation model explained 4.9% of the variance in psychological well-being, 14.1% of the variance in self-confidence, and 15.8% of the variance in motivation. Notably, the inclusion of psychological well-being and self-confidence as serial mediators increased the explained variance in motivation from 2.9% (in the model with intrinsic religious motivation as the sole predictor) to 15.8%. The incremental contribution of psychological well-being corresponded to a small-to-medium effect size (Cohen’s *f*^2^ = 0.100), while the incremental contribution of self-confidence corresponded to a small but significant effect size (Cohen’s *f*^2^ = 0.020). Although these effect sizes are statistically significant, their practical and performance-level implications should be interpreted with caution. Small statistical effects may not translate into meaningful contributions to athlete performance in the field, or they may necessitate different prioritization in the design of intervention programs. These findings underscore the importance of examining mediating mechanisms rather than relying solely on direct predictor–outcome relationships. The results carry several tentative practical implications, which should be interpreted in light of the cross-sectional design. Although athletic performance was not directly measured in this study, the observed associations suggest that sport psychologists and mental performance consultants may consider meaning-oriented and holistic approaches that integrate athletes’ individual values and belief systems to support their psychological well-being. Considering religiosity-based spirituality as a complementary element supporting athletes’ psychological health may inform more holistic policies for athlete well-being, though the link to on-field performance outcomes remains to be established through longitudinal and intervention-based research. It is worth noting the magnitude of these associations. IRM explained approximately 5% of the variance in PWB (*R*^2^ = 0.049, Cohen’s *f*^2^ = 0.10), representing a small-to-medium effect. While this aligns with the expectation that religiosity is one of many contributors to well-being, the serial indirect pathway (IRM → PWB → SC → MOT) remained statistically significant despite these modest individual links, suggesting a cumulative indirect pathway. The sample’s Muslim-majority composition, drawn from a Turkish context where religious practice is culturally embedded, may partly explain the stronger PWB pathway relative to findings in more secular samples. Future research in religiously diverse populations would help clarify the generalizability of these patterns.

### Limitations and Future Research

Several limitations of the present study should be acknowledged. First, the cross-sectional research design does not permit causal inferences; the directed paths in the serial mediation model reflect theoretical assumptions rather than established temporal sequences. Longitudinal or experimental designs are required to confirm the proposed causal chain. Second, the restriction of the sample to a specific cultural and athletic context within Türkiye, combined with the use of convenience sampling, limits the representativeness and generalizability of the findings. Moreover, the demographic information form collected only age and gender data; detailed athletic characteristics such as sport type (individual vs. team sports), competitive level, and years of competitive experience were not systematically recorded, which precludes more nuanced subgroup analyses. Additionally, although the study was conducted in Türkiye, a predominantly Muslim-majority country, religious affiliation was not formally assessed. The sample likely comprises predominantly Muslim participants, which limits the generalizability of the intrinsic religious motivation findings to religiously diverse populations. Third, the operationalization of spirituality as a unidimensional construct (intrinsic religious motivation) constrains the examination of its different dimensions. Fourth, some effect sizes are modest (*R*^2^ = 0.049 for psychological well-being), suggesting that intrinsic religious motivation explains only a small proportion of variance and that unmeasured variables likely contribute to these outcomes. Fifth, all variables were measured simultaneously using self-report instruments, introducing the risk of common method variance. Although Harman’s single-factor test and CFA results suggested that common method variance did not pose a serious threat, these procedures do not provide definitive assurance. Sixth, although data were collected through both online (*n* = 278, 69.3%) and face-to-face (*n* = 123, 30.7%) administration, the mode of administration was not recorded as a participant-level variable during data collection. Consequently, a direct statistical comparison of responses across administration modes could not be conducted. However, both modes used identical instruments with standardized instructions, and prior research has demonstrated measurement equivalence of self-report psychological scales across online and paper-based formats (Weigold et al., 2013). Seventh, the convergent validity of the measurement instruments, as assessed by AVE, fell below the conventional 0.50 threshold for three of the four scales (IRM = 0.42, PWB = 0.40, SC = 0.30), while the Motivation scale met the threshold (MOT = 0.52). Although Composite Reliability values exceeded 0.70 for all scales, supporting adequate construct-level reliability, the below-threshold AVE values indicate that a substantial proportion of item-level variance was not captured by the latent factors. This is particularly noteworthy for the Self-Confidence Scale (AVE = 0.30), where only 30% of the item-level variance is attributable to the intended construct; the remaining variance reflects measurement error or construct-irrelevant sources. Low AVE reduces confidence that the composite scores fully represent the intended latent constructs, which in turn introduces uncertainty into the path coefficients derived from those composites. In observed-variable path analysis, this measurement imprecision is absorbed into the composite scores and can bias path coefficients in either direction—not necessarily toward attenuation—depending on the model configuration and inter-variable correlation patterns (Cole & Preacher, 2014). The path analysis results should therefore be interpreted with this measurement limitation in mind. This imprecision may be partly attributable to method effects associated with reverse-scored items and the use of brief adapted scales (DiStefano & Motl, 2006). Future research should consider latent-variable structural equation modeling, which explicitly separates measurement error from true score variance, to provide more precise estimates of the structural relationships.

Finally, the ordering of variables in cross-sectional data is based on theoretical assumptions; alternative mediation orderings may yield equivalent statistical fit, and comparative testing of alternative models would strengthen the robustness of the findings.

Future research should employ longitudinal designs with multiple measurement points, work with diverse samples across different cultural contexts, include explicit measures of religious affiliation, consider the multidimensional nature of spirituality, pursue item-level refinement to improve convergent validity, and incorporate additional potential mediating or moderating variables such as coach–athlete relationship quality, perceived social support, and training experience. The present study employed observed-variable path analysis with composite scores rather than latent-variable structural equation modeling. Given that AVE values were below 0.50 for three of four constructs, future research should consider latent-variable SEM to account for measurement error and provide a robustness check.

## Figures and Tables

**Table 1:** Descriptive Statistics, Reliability Indices, and Correlations Among Study Variables.

Variable	*M*	*SD*	Skew.	Kurt.	α	ω	AVE	CR	1	2	3	4
1. IRM	39.31	6.08	−0.17	−0.43	0.82	0.85	0.42	0.87	—			
2. PWB	45.17	4.80	−0.59	−0.97	0.77	0.78	0.40	0.84	0.22 **	—		
3. SC	51.67	5.99	−0.27	−0.25	0.70	0.69	0.30	0.79	0.28 **	0.30 **	—	
4. MOT	16.19	2.15	−0.12	0.01	0.67	0.64	0.52	0.80	0.17 **	0.36 **	0.25 **	—

*Note. N* = 401. IRM = Intrinsic Religious Motivation; PWB = Psychological Well-Being; SC = Self-Confidence; MOT = Motivation. α = Cronbach’s alpha; ω = McDonald’s omega; AVE = average variance extracted; CR = composite reliability. Skew. = skewness; Kurt. = kurtosis. Correlation coefficients are Pearson’s *r*. ** ***p* < 0.01.**

**Table 2:** Confirmatory Factor Analysis (CFA) Fit Indices for the Study Scales.

Scale	c^2^/df	*p*-Value	GFI	CFI	RMSEA	IFI	PNFI
**Acceptance Criteria**	≤5	<0.05	≥0.90	≥0.90	≤0.08	≥0.90	>0.50
Intrinsic Religious Motivation	3.31	0.001	0.99	0.96	0.07	0.96	0.64
Psychological Well-Being	3.10	0.001	0.98	0.94	0.07	0.94	0.62
Self-Confidence	2.80	0.001	0.87	0.90	0.05	0.90	0.78
Motivation	2.00	0.0368	1.00	1.00	0.02	1.00	0.33

Note: Model, χ^2^/df = Chi-Square/Degrees of Freedom, RMSEA = Root Mean Square Error of Approximation, NFI = Normed Fit Index, GFI = Goodness of Fit Index, CFI = Comparative Fit Index., IFI = Incremental Fit Index, PNF = Parsimony Normed Fit Index (Mishra & Datta, 2011; Schreiber et al., 2006).

**Table 3:** Summary of Hypothesis Testing Results.

	Path	B	SE	β	*t*/95% CI	*p*	Result
Direct Effect Hypotheses							
H_1_	IRM → PWB	0.218	0.048	0.221 **	4.51	<0.001	Supported
H_2_	IRM → SC	0.205	0.043	0.229 **	4.80	<0.001	Supported
H_3_	PWB → SC	0.228	0.043	0.252 **	5.28	<0.001	Supported
H_4_	PWB → MOT	0.275	0.044	0.307 **	6.29	<0.001	Supported
H_5_	SC → MOT	0.140	0.049	0.141 **	2.84	<0.005	Supported
Total effect (c):	IRM → MOT	0.152	0.044	0.171 **	3.47	<0.001	
Direct effect (c′)	IRM → MOT	0.056	0.043	0.063	1.30	0.194	
Mediation Hypotheses							
H_6_	IRM → PWB → MOT	0.060	0.018	0.068 **	0.028	0.098	Supported
H_7_	IRM → SC → MOT	0.029	0.013	0.032 *	0.006	0.057	Supported
H_8_	IRM → PWB → SC → MOT	0.007	0.004	0.008 *	0.001	0.016	Supported

Note. *N* = 401. Direct effect hypotheses (H_1_–H_5_) and total/direct effects (c, c′) report unstandardized coefficients (B), OLS standard errors, and *t*-values. Mediation hypotheses (H_6_–H_8_) report bootstrap standard errors and bias-corrected 95% confidence intervals based on 5000 resamples; mediation is significant when the confidence interval does not contain zero. Unstandardized coefficients (B) and standard errors are reported on the item-mean metric (i.e., scale totals divided by the number of items), so that B reflects the expected change in the outcome per one-unit change on the Likert response scale. IRM = Intrinsic Religious Motivation; PWB = Psychological Well-Being; SC = Self-Confidence; MOT = Motivation. LLCI = lower limit; ULCI = upper limit of 95% confidence interval. The serial mediation model was tested using Hayes’s (2022) PROCESS macro (Model 6). * *p* < 0.05. ** *p* < 0.01. Item-mean standard deviations used for standardization: IRM = 0.61, PWB = 0.60, SC = 0.55, MOT = 0.54.

## Data Availability

The data presented in this study are available on request from the corresponding author due to ethical and privacy restrictions.
